# Transcriptomic and Lipidomic Analysis of Lipids in *Forsythia suspensa*

**DOI:** 10.3389/fgene.2021.758326

**Published:** 2021-10-26

**Authors:** Bei Wu, Yinping Li, Wenjia Zhao, Zhiqiang Meng, Wen Ji, Chen Wang

**Affiliations:** ^1^ Department of Pharmacy, Second Hospital of Shanxi Medical University, Taiyuan, China; ^2^ Institute of Pomology, Chinese Academy of Agricultural, Sciences (CAAS), Xingcheng, China; ^3^ College of Agriculture, Shanxi Agricultural University, Taigu, China; ^4^ Department of Pharmacy, Shanxi Eye Hospital, Taiyuan, China

**Keywords:** Forsythiae Fructus, RNA sequencing, lipid, metabolism, transcriptome, candidate genes, terpenoids, falvonoids

## Abstract

Forsythiae Fructus (Lianqiao in Chinese) is widely used in traditional Chinese medicine. The lipid components in Forsythiae Fructu*s* are the basis of plant growth and active metabolism. Samples were collected at two growth stages for a comprehensive study. Transcriptome and lipidomics were performed by using the RNA-seq and UPLC-Q-TOF-MS techniques separately. For the first time, it was reported that there were 5802 lipid components in Lianqiao comprised of 31.7% glycerolipids, 16.57% phospholipids, 13.18% sphingolipids, and 10.54% fatty acids. Lipid components such as terpenes and flavonoids have pharmacological activity, but their content was low. Among these lipids which were isolated from Forsythiae Fructus, 139 showed significant differences from the May and July harvest periods. The lipids of natural products are mainly concentrated in pregnenolones and polyvinyl lipids. RNA-Seq analysis revealed 92,294 unigenes, and 1533 of these were differentially expressed. There were 551 differential genes enriched in 119 KEGG pathways. The *de novo* synthesis pathways of terpenoids and flavonoids were explored. Combined with the results of lipidomics and transcriptomics, it is hypothesized that in the synthesis of abscisic acid, a terpenoid, may be under the dynamic regulation of genes EC: 1.1.1.288, EC: 1.14.14.137 and EC: 1.13.11.51 in balanced state. In the synthesis of gibberellin, GA20-oxidase (GA20ox, EC: 1.14.11.12), and GA3-oxidase (GA3ox, EC: 1.14.11.15) catalyze the production of active GAs, and EC: 1.14.11.13 is the metabolic enzymes of active GAs. In the synthesis of flavonoids, MF (multifunctional), PAL (phenylalanine ammonia-lyase), CHS (chalcone synthase), ANS (anthocyanidin synthase), FLS (flavonol synthase) are all key enzymes. The results of the present study provide valuable reference information for further research on the metabolic pathways of the secondary metabolites of *Forsythia suspensa.*

## Introduction

*Forsythia suspensa* (Thunb.) Vahl, a plant in the family Oleaceae, is widely planted in East Asia and is a traditional medicinal tree species. It is one of the 40 commonly used medicinal species in China (Sun et al., 2018). Forsythiae Fructus (FF, called Lianqiao in Chinese) is the dried fruit of *F. suspensa,* and in traditional Chinese medicine it is thought to have “heat clearing” and “detoxifying” effects. Currently, FF is used as a medical treatment for pyrexia, inflammation, gonorrhea, carbuncles and erysipelas. Due to different harvest times and processing methods, FF can be divided into “Qingqiao” and “Laoqiao”. Clinically, “Qingqiao” is used more frequently (Guo et al., 2007; Wu et al., 2019). At present, 321 chemical components have been isolated from FF, including phenylethanoid glycosides, lignans, flavonoids, terpenes and volatile oils (Dong et al., 2017). Modern Chinese Medicine studies have found that most of these substances have pharmacological activity (Wang et al., 2018). Forsythiaside A is a phenylethanoid glycoside and it shows remarkable anti-inflammation, antivirus, neuroprotection, antioxidant, hepatoprotection, and antibacterial activity (Gong et al., 2021). Forsythin, a lignan substance, mitigates apoptosis and oxidative stress (Du et al., 2020), has anti-viral and anti-inflammatory activity (Ma et al., 2020), and improves insulin resistance (Xu et al., 2019). Rutin is a flavonoid compound with many pharmacological effects, including antioxidant, anti-inflammatory, anti-diabetic, neuroprotective (Chua, 2013), vascular protection (Stanely and Priya, 2010), and anti-cancer (Ben et al., 2016). The volatile oil of FF is a primary activeone of the main pharmacological components and has the effects of antiviral, antibacterial, antioxidant (Jiao et al., 2012), anti-inflammatory, anti-tumor, acaricidal (Lee and Lee, 2015). The basis of the heat-clearing effect of FF in Chinese medicine prescriptions is the anti-inflammatory and antioxidant properties. The detoxification effect is attributed to its antibacterial, antiviral and anticancer activities (Wang et al., 2018). Based on this, FF is the main active ingredient in many widely used classic Chinese patent medicine prescriptions, such as Shuanghuanglian injections (Zhou et al., 2011) and Lianhua Qingwen granules (Hu et al., 2021). Lianhua Qingwen is also recommended for the treatment of COVID 19 (Hu et al., 2021; Liu et al., 2021). Because FF is widely used, it is necessary to conduct research on this product and its pharmacologically active substances.

The pharmacologically active substances in FF are secondary metabolites. Plant growth, development and reproduction are achieved through material metabolism. Secondary metabolism produces compounds that are often involved in ecological interactions and include pigments, scents, and antibiotics. Secondary metabolites are used in medicine, food, dyes, and agriculture (Srivastava and Srivastava, 2007). Plant-derived medicinal secondary metabolites are mainly obtained by direct extraction from plants. However, to produce such compounds on a large scale would require medicinal crops to be grown extensively and most are currently not well suited to production agriculture. Also, the content of medicinal secondary metabolites in plants is low and extraction and separation are difficult. New production methods are being adopted, including extraction from plant tissue cell culture and the use of microbial cell factories for production (Jiang et al., 2021). The use of artificial microbial cell factories is considered feasible, and this approach has been used for the antimalarial drug artemisinin (Ro et al., 2006), the analgesic drug morphine (Thodey et al., 2014), the antitumor drug paclitaxel (Chen et al., 2019), the cardiovascular disease drug tanshinone (Yadav et al., 2020), and other aromatic secondary products. Efficient synthesis of metabolites is an important prerequisite for constructing a microbial cell factory for the secondary metabolites of FF and realizing efficient utilization of FF. The biosynthetic pathway analysis of secondary metabolites is an important prerequisite to improved production methods. Jiao et al. (2013) found that the oil yield of FF’s seeds is about 30% and showed that FF seeds have great potential as a biodiesel raw material resource. FF’s contain volatile oils, flavonols, anthocyanins, β-pinene and other compounds. The synthesis and metabolism of secondary metabolites are mostly related to lipids. Abscisic acid (ABA) is insoluble in water. As a plant hormone, it has physiological functions that include causing bud dormancy, leaf shedding, and inhibiting cell growth, and ABA can promote flavonoid metabolism. Lipid metabolism is a key process in energy homeostasis, carbon storage, membrane structure, cell signaling, transcription and translation regulation, and cell and protein interactions (Chapman and Feussner, 2016). Therefore, lipids play an important role in plant growth, development, and reproduction (Chapman and Feussner, 2016). Lipids are unique and functionally specific compared to other metabolites. Therefore, lipid composition and abundance can be used to monitor changes in plants over time and their response to specific stimuli. The lipid components of FF have not been fully studied. Because of the importance of these compounds, a comprehensive analysis and comparison of them in FF is needed. Clarifying the biosynthetic pathways and regulation methods of secondary metabolites in FF will be useful for stabilizing and improving this important product. The content of secondary metabolites, the improvement of the quality of medicinal materials, and the biological preparation technology of secondary metabolites are of great significance. Lipidomics deals with lipids and lipid interactions in biological systems. Lipidomic analysis methods include chromatography, nuclear magnetic resonance spectroscopy, and mass spectrometry-based analysis methods. Liquid chromatography-mass spectrometry is currently the most widely used method for lipidomics research. Chromatography mass spectrometry uses the separation ability of chromatography and the high sensitivity of mass spectrometry to achieve in-depth analysis of lipids (Xu et al., 2020). To identify and quantify all lipid components in FF and systematically analyze the lipid composition and expression changes in the growth process of FF, the present study applied high-resolution liquid phase tandem mass spectrometry technology for lipidomic analysis.

The syntheses of secondary metabolites requires the participation of various metabolic pathways, and is regulated by gene expression. High-throughput transcriptome sequencing analysis can reflect the gene expression of cells, tissues, or organisms over time and can identify candidate genes related to the metabolism of active substances. This analysis method provides gene regulation clues for the biosynthetic analysis of FF secondary metabolites. This study makes use of transcriptomics and lipidomics methods to 1) determine the lipid composition of FF over time during normal growth, and 2) screen candidate genes related to the synthesis of lipid secondary metabolites. Results can provide a reference for increasing the content of secondary metabolites in FF, breeding high-quality FF varieties, and realizing large-scale production of secondary metabolites.

## Materials and Methods

### Plant Material and Treatments

**FIGURE 1:**
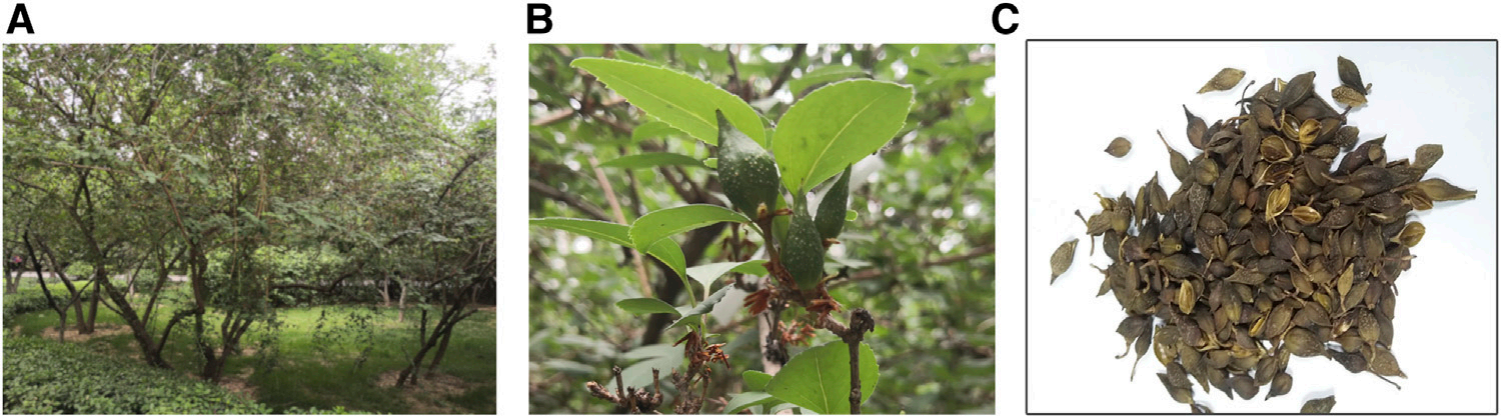
**(A)** *Forsythia suspensa* (Thunb.) Vahl; **(B)** *Forsythia* fruit growing on the tree **(C)** Forsythiae Fructus as a medicinal material.

FF was harvested in May and July 2021 in Taiyuan, Shanxi (112°33′E, 37°54′N, elevation 800 m, Figure 1). Normal plants were selected. Three to five fruits were taken from the same plant, frozen in liquid nitrogen for 120 min immediately after harvesting, and stored in an ultra-low temperature refrigerator at −80°C. The samples were similar in size and were identified by Jinping Luo, Chief Pharmacist of the Institute of Pharmaceutical Inspection Technology, Shanxi Provincial Inspection and Testing Center. The specimens were stored in the ultra-low temperature refrigerator at −80°C in the laboratory of the Department of Pharmacy, Second Hospital of Shanxi Medical University.

### Chemicals and Reagents

Methanol: 67-56-1, LC-MS, Merck; Acetonitrile: 75-05-8, LC-MS, Merck; Ammonium acetate: 631-61-8; Formic acid: 64-18-6, LC-MS, TCI; Isopropanol: 67-63-0, LC-MS, Merck; Methyl tert-butyl ether, TCI LC-MS, TCI; Methyl tert-butyl ether (MTBE): 1634-04-4, LC-MS, Merck; Dichloromethane: 75-09-2, LC-MS, Merck.

### Sample Preparation and Extraction

FF picked in May were designated as group A and those picked in July were designated as group B. Three samples of each group of the same size were selected. The sample is ground into powder with liquid nitrogen. A sample of 50 mg each were weighed, 200 μL of water was added, and the samples vortexed for 30 s. Steel beads were added, and the samples were ground at 1000 rpm for 12 min, then sonicated for 15 min in an ice water bath. Next, 480 μL of extraction solution (methyl tert-butyl ether: methanol = 5:1) was added, the samples vortexed for 30 s, sonicated for 10min in an ice-water bath, and allowed to stand for 1 hour at minus 20°C. The samples then were centrifuged at 10,000 rpm for 15 min at 4°C. A supernatant alloquot of 380 μL was removed, dried under vacuum, and 200 μL of 1:1methylene chloride: methanol was added for re-solubilization, the solution vortexed for 30 s, and sonicated for 10 min in an ice-water bath. The samples were centrifuged at 13,000 rpm for 15 min at 4°C. From each sample, 180 μL of supernatant wasremoved and mixed into a quality control sample by taking 10 μL of each sample.

### Chromatographic Conditions

The sample extracts were analyzed using a UPLC Acquity I-Class PLUS column (Waters). The UPLC column analytical conditions were as follows. Waters Acquity UPLC CSH C18 (1.7 μm, 2.1 mm, 100 mm); injection volume 5 μL; temperature 55°C; flow rate 400 μL/min; mobile phase A 60% acetonitrile aqueous solution, 10 mM ammonium acetate, 0.1% formic acid; mobile phase B 90% isopropanol acetonitrile solution, 10 mM ammonium acetate, 0.1% formic acid, 10 mM ammonium acetate, 0.1% formic acid. Gradient elution parameters: 0.0–2.0 min, 60–57% A, 40–43% B; 2.1–12.0 min, 50–46% A, 50–54% B; 12.1–18.0 min, 30–1% A, 70–99% B; 18.1–20.0 min, 60% A, 40% B.

### Mass Spectrometry Conditions

After UHPLC separation, mass spectrometry was performed with a UPLC Xevo G2-XS QT of (Waters) with electrospray ionization (ESI) positive and negative ion mode detection. ESI ion source parameters were as follows: capillary voltage 2000 V (positive ion mode) or −1500 V (negative ion mode); cone hole voltage: 30 V; ion source temperature: 120°C; dissolvent gas temperature 550°C; backblast gas flow rate: 50 L/h; dissolvent gas flow rate: 900 L/h. The MSe flow rate was controlled by MassLynx V4.2 (Waters). Primary and secondary mass spectrometry data acquisition was performed using MSe mode under MassLynx V4.2 (Waters) control. In each data acquisition cycle, dual-channel data acquisition was performed simultaneously for low and high collision energies. The low collision energywas 2 V, and the high collision energy range was 10–40 V. The scan frequency was 0.2 s for one mass spectrometry map.

### Lipid Identification and Quantification

Lipid molecules were identified by referring to the LIPID MAPS (http://www.lipidmaps.org/) and Lipid Bank (http://www.lipidbank.jp/) lipid databases, combined with data on retention times of chromatograms in positive and negative ion modes, primary mass spectra and secondary mass spectra.

The statistical method of orthogonal partial least squares-discriminant analysis (OPLS-DA) was used to analyze non-orthogonal and orthogonal variables separately to obtain more reliable intergroup differences in lipids concerning the degree of correlation between experimental groups information. OPLS-DA model calculations were performed mainly using the R (3.3.2) package ropls. The prediction parameters of the evaluation model are R2X, R2Y, and Q2, where R2X and R2Y indicate the explanation rate of the built model for X and Y matrices, respectively, where the X matrix is the model input, i.e. the lipid quantification matrix, and the Y matrix is the model output, i.e., the sample grouping matrix, and Q2 indicates the prediction ability of the model, i.e., whether the built model can distinguish the correct sample grouping by metabolic expressions. The closer R2Y and Q2 are to 1, the more stable and reliable is the model for screening differential lipids. In general, a model with Q2 > 0.5 can be considered valid, and a model with Q2 > 0.9 is an excellent model.

The lipidomics data were analyzed for lipid fraction differences mainly by clustering heat map analysis and difference multiplier analysis. Differential lipids were screened by combining the difference multiples, *p*-value of a *t*-test, and VIP value of the OPLS-DA model. The screening criteria were Fold Change (FC) > 2, *p*-value < 0.05 and VIP >1.

### RNA Extraction, Sequencing, and *de novo* Assembly

Forsythiae Fructus were sampled for transcriptome sequencing experiments. Sequencing libraries were generated using NEBNext®Ultra™ RNA Library Prep Kit for Illumina® (NEB, United States) following the manufacturer’s recommendations and index codes were added to attribute sequences to each sample. The mRNA was purified from total RNA using poly-T oligo-attached magnetic beads. Fragmentation was done using divalent cations under elevated temperature in NEBNext First Strand Synthesis Reaction Buffer (5X). First strand cDNA was synthesized using random hexamerprimer and M-MuLV Reverse transcriptase. Second strand cDNA synthesis was subsequently performed using DNA polymerase I and rnase H. Fragments of cDNA 240bp in lengthpreferentially selected. The clustering of the index-coded samples was performed on a cBotCluster Generation System using the TruSeq PE Cluster Kit v3-cBot-HS (Illumia). The library preparations were sequenced on an Illumina Hiseq (6,000 platform and paired-end reads were generated. About 6 GB of raw data from each sample were sequenced. Clean data were obtained by removing reads containing the adapter, reads containing ploy-N, and low quality reads from raw data. Clean data analysis was performed using Trinity.

### Gene Annotation and Analysis of Differential Genes

Gene function was annotated based on the following databases: NR (NCBI nonredundant protein sequences), Pfam (Protein family), KOG/COG (Clusters of Orthologous Groups of Proteins), eggNOG (evolutionary genealogy of genes: Non-supervised Orthologous Groups), Swiss-Prot (a manually annotated and reviewed protein sequence database), KEGG (Kyoto Encyclopedia of Genes and Genomes), and GO (Gene Ontology). Picard - tools v1. 41 and samtools v0. 1.18 were used to sort, remove duplicated reads and merge the bam alignment results of each sample. Gene expression levels were estimated by RSEM for each sample. Fragments per kilobase of transcript per million mapped reads (FPKM) were used to indicate the abundance of expression of the corresponding unigene.

Differential expression analysis of two conditions/groups was performed using the DESeq R package (1.10.1). DESeq provides statistical routines for determining differential expression in digital gene expression data using a model based on the negative binomial distribution. The resulting *p*-values were adjusted using the Benjamini and Hochberg’s approach for controlling the false discovery rate (FDR). The *p*-value was adjusted using the q-value, which was set as the threshold for significantly differential expression. The differentially expressed genes (FDR < 0.01, FC > 2) were analyzed.

GO enrichment analysis of the differentially expressed genes (DEGs) was implemented by the top GO R packages-based Kolmogorov–Smirnov test. We used KOBAS software to test the statistical enrichment of differentially expressed genes in KEGG pathways.

The lipidome and transcriptome data of FF were fitted to analyze the regulation mechanism of lipid secondary metabolite synthesis, and candidate genes that affect the synthesis pathway were screened. According to reports, the pharmacologically active lipid secondary metabolites in FF are mainly flavonoids and terpenes. The presentstudy focuses on the analysis of these two types of compounds.

## Results

### Data Quality Evaluation

**FIGURE 2:**
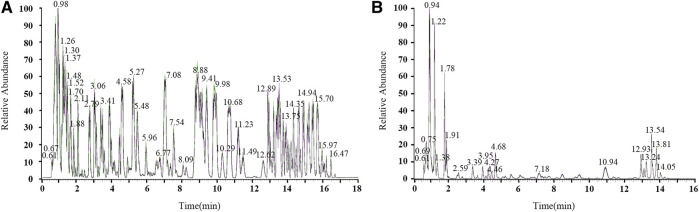
Chromatogram of the quality control sample in ESI-positive **(A)** and ESI-negative **(B)** ion modes.

Before the formal UHPLC-Q-Tof-MS experiment, the base peak chromatogram of the quality control sample was compared for spectral overlap. The peak response intensities and retention times of the samples overlapped, indicating the experimental instrument has excellent stability and reproducibility (Figure 2).

Transcriptomics experiments were grouped in the same way as lipidomics experiments. The Spearman’s rank correlation coefficient (r) was used as an index to assess the correlation between sample groups in the transcriptomics experiments. When *r* ^2^ is closer to 1, a strong correlation between the two samples is indicated. The intra-group sample correlation *r* ^2^ > 0.7 is shown in the attached Supplementary Figure S1.

### Lipid Composition and Content

**FIGURE 3:**
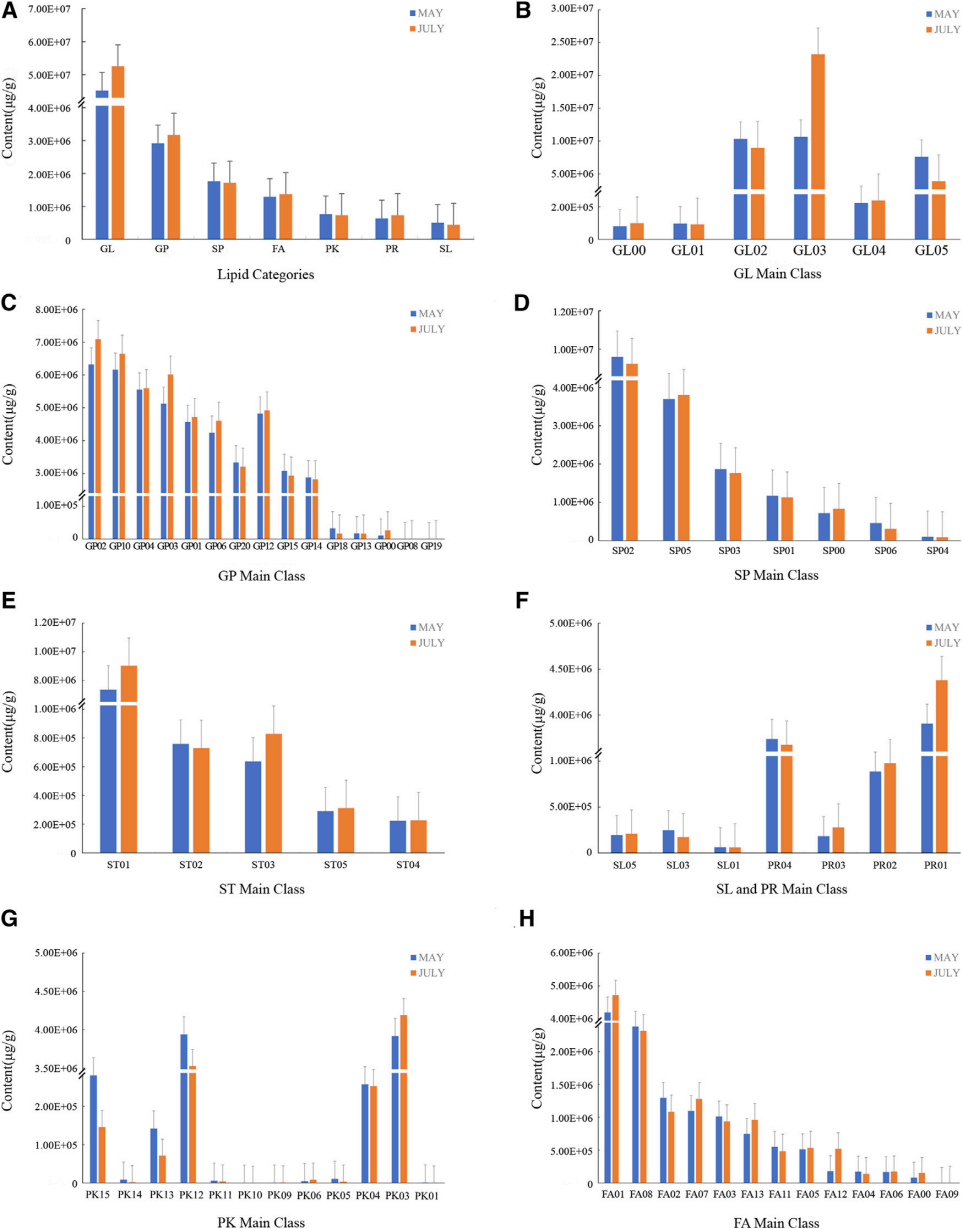
The content of various lipid components of FF differed with growth stage. The May sample is represented in blue and the July sample in red. For each sample date, **(A)** The composition of total lipids contained in FF, **(B)** Glycerolipids (GL), **(C)** Glyceropnospholipids (GP), **(D)** Sphingolipids (SP), **(E)** Sterol Lipids (SL), **(F)** Saccharopilipids (SL) and Prenol Lipids (PR), **(G)** Polyketides (PK), **(H)** Fatty Acyls (FA).

According to the LMSD (LIPID MAPS Structure database, http://www.lipidmaps.org/data/structure) database classification, 8categories, 66 main classes, 257 lipid subclasses, and 5,802 lipid molecules were detected in FF (Figure 3). Glycerolipids (GL) were the most abundant, with 6 main classes and 640 lipids. GLaccounted for about 29.55 and 31.70% of the lipids in group A and B,respectively. Between the two sampling dates, the content of triacylglycerol increased significantly, and the content of dialkylglycerol and glycosyl dihexylglycerol decreased. Glycerophospholipids (GP) were the next most abundant, with 15 main classes and 931 lipid substances, with 29, 163, 738.21 μg/g and 31, 718, 781.85 μg/g in groups A and B, respectively, accounting for about 16.13 and 16.57% of the total lipids. Across sampling dates, the contents of glycerophosphocholine (PC), glycerophosphoethanolamine (PE), glycerophosphoserine (PS), glycerophosphoinositol (PI) and glycerophosphate (PA) all increased and the content of oxidized glycerophospholipids decreased significantly. Sphingolipids (SP) ranked third, with seven main classes and 726 lipid substances, and the contents of the A and B groups were 17, 631, 677.45 μg/g and 17, 180, 898.39 μg/g, respectively, accounting for about 14.77 and 13.18%. Ceramide content increased significantly from group A to B, phospholipids and acid glycosphingolipids decreased slightly, and neutral glycosphingolipids and other sphingolipids increased slightly. Fatty acyles (FA) had 13 main classes and 1,695 lipid substances and the contents of the two groups were 12, 881, 185.91 μg/g and 13, 739, 216.99 μg/g, accounting for about 10.79 and 10.54% of total lipids. From group A to B, the content of fatty acids and their conjugates, fatty esters, oxygen-containing hydrocarbons, fatty acyl glycosides and other fatty acyl groups increased whereas the higher fatty acids (octadecane, eicosane, behenyl) and hydrocarbons decreased. There were five main classes of sterol lipids (ST) and 771 lipids with 9,870,796.40 μg/g and 11, 216, 784.53 μg/g, respectively, accounting for 8.27 and 8.61% of total lipids. There were 12 main classes of polyketides (PK) with 544 lipids, with 7,658,121.17 μg/g and 7,343,043.54 μg/g, accounting for 6.42 and 5.63% of total lipids, respectively. In PK, the content of acetone in Annonaceae increased; the content of flavonoids and phenolic esters decreased. There were four main classes of prenol lipids (PR) with 443 lipids, with 6,366,599.90 μg/g and 7,369,347.98 μg/g, accounting for 5.33 and 5.65% of total lipids, respectively. The content of isoprenoids, polypentenols, quinones and hydroquinones increased across sampling dates. Among these compounds were limonene-1,2-diol and (+)-exo-5-hydroxycamphor monoterpenes, selina-4 (14), 7,11-trien-9-ol and other sesquiterpenes, (-)-enunicelline diterpenes such as ximaosarcophytol A, triterpenes such as squalen-1-ol and epi-maslinic acid, and tetraterpenes such as amarouciaxanthin B/sidnyaxanthin and diatoxanthin/7,8-didehydrozeaxanthin. There were also gibberellin (GA) (including GA_36_, GA_8_, GA_9_, GA_24_, GA_17_, GA_7_), abscisic acid, zeatin, lutein, and other plant hormones, but their content did not differ between groups A and B. There were three main classes of glycolipids (SL) with 52 lipids, the contents of groups A and B were 505,912.76 μg/g and 442,897.79 μg/g accounting for 0.42 and 0.34% of total lipids, respectively. The total contents of various other types of lipids in the two groups of samples were less varied between groups A and B.

### Differential Lipid Composition

**FIGURE 4:**
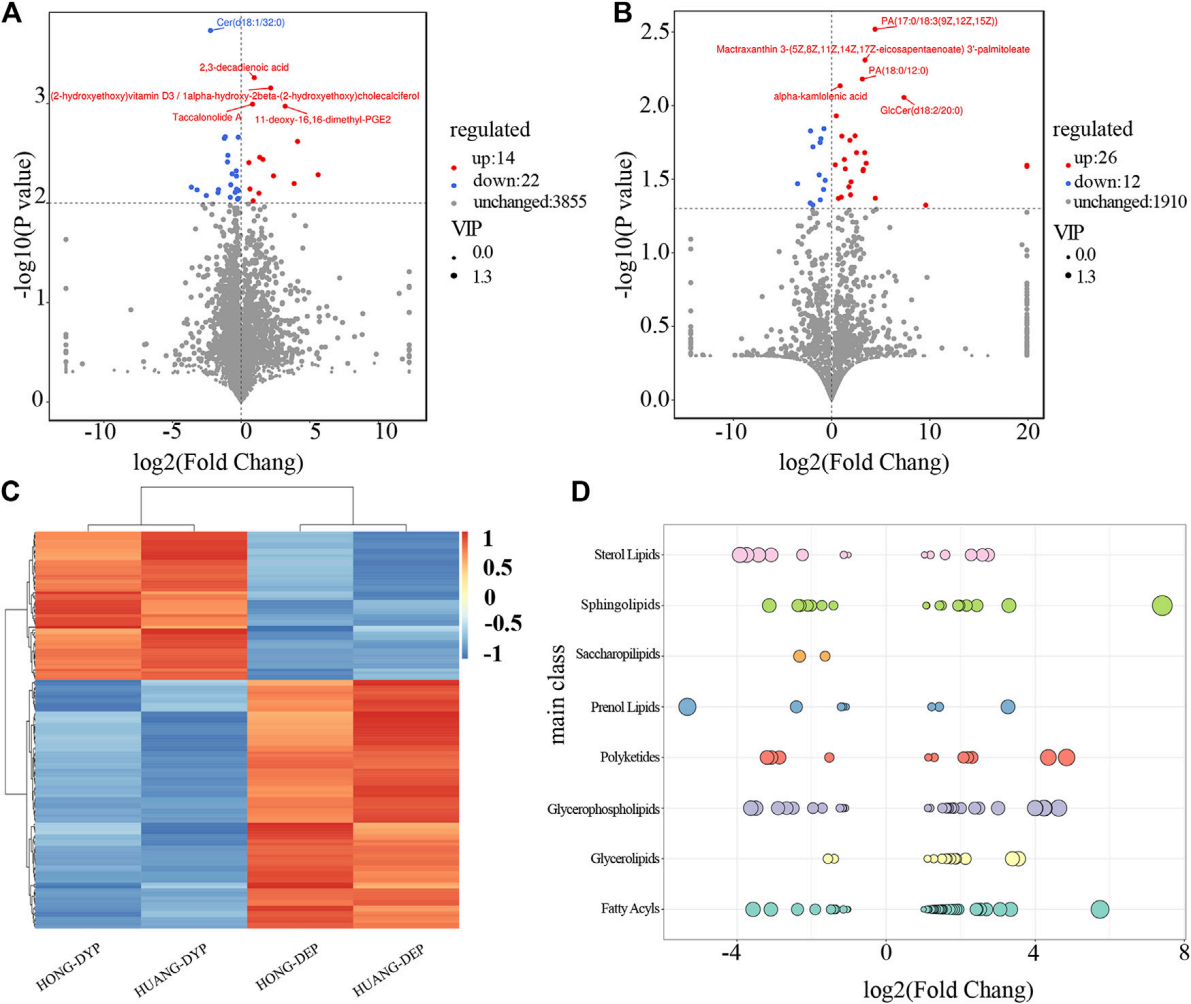
Analysis of differential lipid molecules in FF from May and July sampling dates. Characteristics of differential lipid molecules of FF in ESI-positive **(A)** and ESI-negative **(B)** ion modes. The dots represent lipid molecules, among which the blue (down regulated) and red dots (up regulated) that satisfied FC < 0.5, FC > 2, and *p* < 0.05 indicating significant differences between the two sample dates **(C)** Hierarchical and clustering analysis **(D)** Representation of significantly different lipid distributions at different stages. Each dot represents a lipid, and the larger the dot, the greater the difference in lipids across sampling dates. Log2FC is the logarithmic value of the lipid content of the two groups. Log2FC > 0 means that the lipid content increased from the May to July sample, and Log2FC < 0 means that the lipid content decreased in from the May to July sample.

The results of the orthogonal partial least-squares discriminant analysis (OPLS-DA) of differences grouping scores for each group are shown in Supplementary Figure S2. Among the two groups (A and B) of samples harvested at different periods, significant differences were considered according to the difference multiplier FC > 2 or FC < 0.5, *p*-value < 0.05 in the *t*-test, and VIP >1 in the OPLS-DA model, which in turn screened for variation differential lipids. A total of 139 lipid molecules were significantly different, accounting for 2.40% of the total number of lipids of which 87 (1.50%) had upregulated expression levels (Figure 4).

FF sampled in May and July had significant differences in the content of phospholipids, fatty acids, glycerolipids, sphingolipids, and sterol lipids. The contents of 38 types of FA were significantly changed, and 27 types (71.05%) were upregulated representing ten subgroups (Figure 4D). Wax monolipids, branched chain fatty acids, prostaglandins, fatty alcohols and hydroxy fatty acids all increased significantly, while rhamnolipids, lactones, and some prostaglandins decreased significantly. The contents of 29 type of GP increased representing seven subgroups, and 17 types (58.62%) were upregulated. The content of GP such as glycerophosphatidylcholine, glycerophosphoglycerol, and glycerophosphoethanolamine increased, while glycerophosphoglycerol and glycerophosphoinositol significantly decreased. The contents of 19 types SP significantly changed, and 11 types (57.89%) were upregulated representing four subgroups. Ceramide increased, and neutral glycosphingolipid substances both increased and decreased. The contents of 15 types of GL significantly changed, and 12 types (80.00%) were upregulated representing four subclasses. The diacylglycerols and triacylglycerols were the largest sub-groups and their content increased from May to July. The monoalkylglycerol subgroup content decreased significantly. The contents of 15 types of ST were significantly changed, and six types (40.00%) representing four subgroups were upregulated. There are mainly sterols present. There were only two different glycolipid components, and the content of the two acyltlehaloses decreased significantly.

PR and PK are the pharmacologically active lipid components of FF. The contents of 12 types of PK were significantly changed, and seven types (58.33%) representing three subclasses were upregulated. Among the PK, chalcone and dihydroxychalcone increased significantly, and flavonoids and flavonols either increased or decreased. The content of nine type PR changed significantly, and they represented three subgroups. Isoprenoids accounted for the main components, mactraxanthin 3-(5Z, 8Z, 11Z, 14Z, 17Z-eicosapentaenoate) 3′-palmitoleate (tetraterpene) and (-)-5-adianene (triterpene) increased, whereas curmadione, (+)-18-hydroxy-7,16-sacculatadiene-11,12-dial, 17β,21β-epoxy-16α-ethoxyhopan-3β-ol, 3-O-acetyl-11-keto-β-boswellic acid and bisdehydro-β-carotene/tetradehydro-β-carotene decreased.

### RNA Sequencing

RNA sequencing-based transcriptome profiling was performed for the FF samples. The minimum ratio of clean data to raw reads was 93.71%. A total of 92,294 single genes with an average length of 787.75 bp and an N50 value of 1,394 bp were botained, of which 20,317 were more than 1 kb in length.

### Functional Annotation and Classification

**FIGURE 5:**
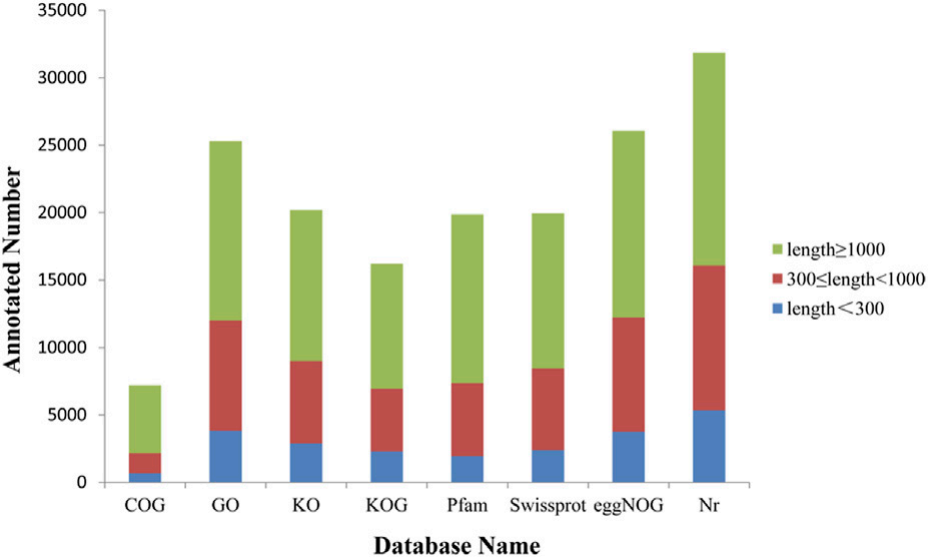
Gene annotations in different databases. The *X*-axis is the database name. The *Y*-axis is the number of genes annotated. Unigenes length≥1000 bp represented in green, unigenes of length 300 bp to 1000 bp represented in red, and unigenes of length ≤300 bp in blue.

The functional annotation of Forsythia unigenes was done using eight databases, including COG, GO, KO, Pfam, Wiss-Prot, eggNOG, NR. The annotation results of 7,202, 25,296, 20,193, 16,213, 19,865, 19,944, 26,079, 31,859 unigenes were obtained respectively, with a total of 32,459 items (Figure 5). Of the total unigenes identified, 3,810 of them had hits in all eight databases.

The sequences were assigned with GO terms based on annotation. Assignment of 25,296 unigenes fell into three GO categories, biological process, molecular function and cellular components. In the KEGG enrichment, 20,193 unigenes were assigned to 136 KEGG pathways. Plant-pathogen interaction (ID: Ko04626), plant hormone signal transduction (ID: Ko04075) and carbon metabolism (ID: Ko01200) were the top 3 pathways with the most assigned unigenes.

### Clustering of Differentially Expressed Gene

**FIGURE 6:**
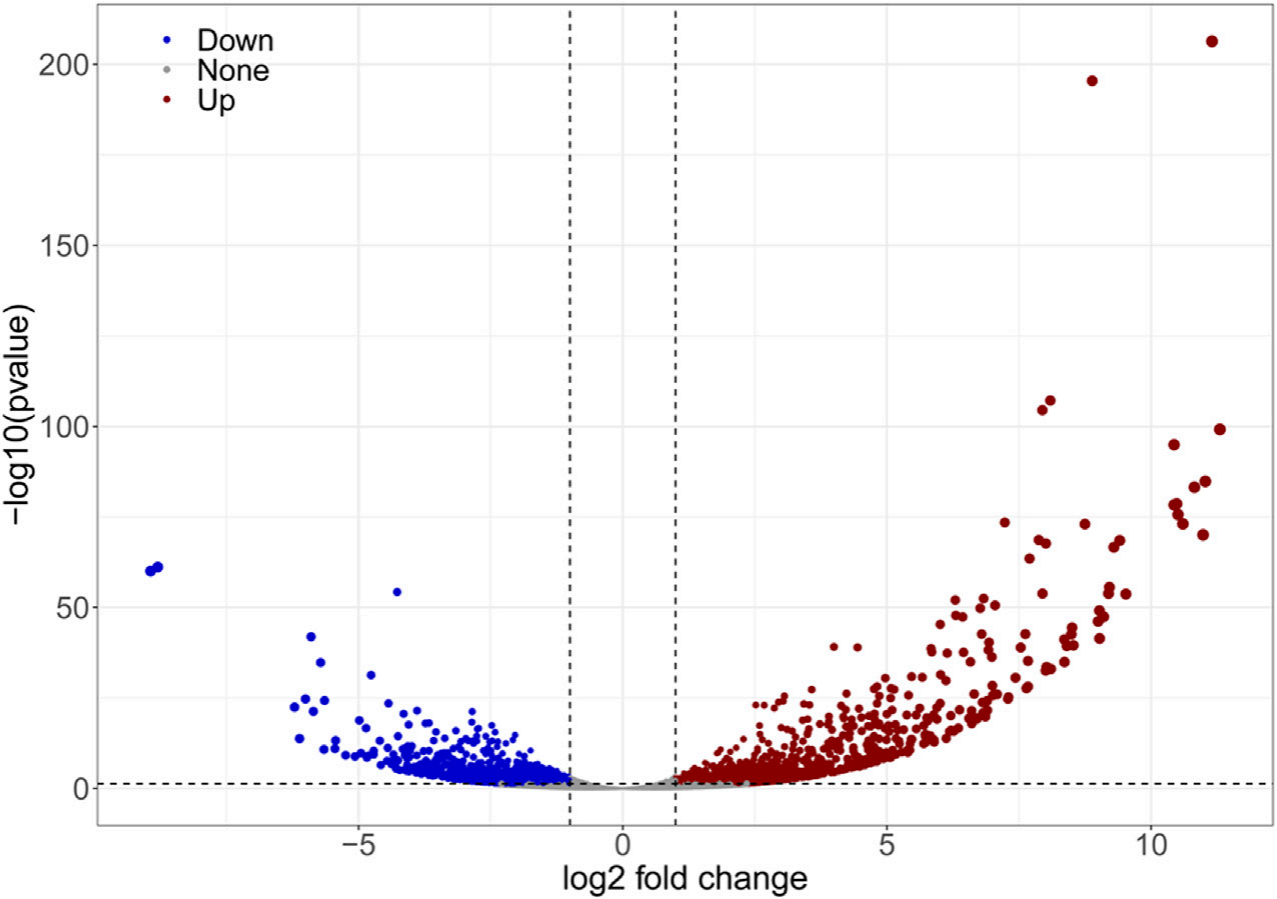
Volcano plot showing the characteristics of differentially expressed gene. The dots represent genes, among which the blue and red dots are genes that satisfied FC < 0.5, FC > 2 and *p* < 0.05 and thus differed in expression between the two samples.

The results of DEGs expression analysis based on mean FPKM showed that 1,533 DEGs were differentially expressed of which 45.27% were upregulated. The degree of difference in gene expression levels between the two groups of samples differed significantly (Figure 6).

**FIGURE 7:**
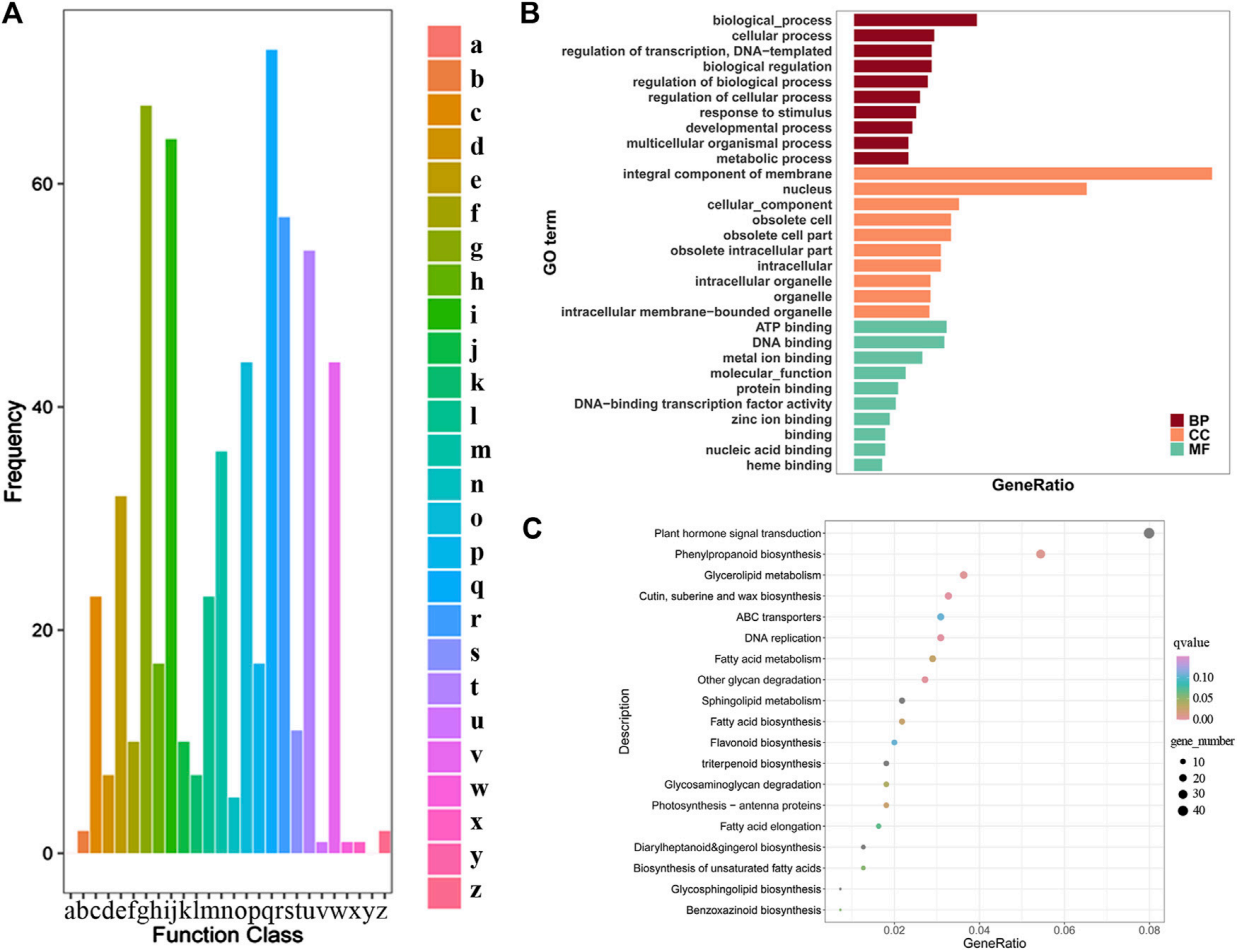
**(A)** COG Function Classification of Consensus Sequence. a) RNA processing and modification; b) Chromatin structure and dynamics; c) Energy production and conversion; d) Cell cycle control, cell division, chromosome partitioning; e) Amino acid transport and metabolism; f) Nucleotide transport and metabolism; g) Carbohydrate transport and metabolism; h) Coenzyme transport and metabolism; i) Lipid transport and metabolism; j) Translation, ribosomal structure and biogenesis; k) Transcription; l) Replication, recombination and repair; m) Cell wall/membrane/envelope biogenesis; n) Cell motility; o) Posttranslational modification, protein turnover, chaperones p) Inorganic ion transport and metabolism; q) Secondary metabolite biosynthesis, transport and catabolism r) General function prediction only; s) Function unknown; t) Signal transduction mechanisms; u) Intracellular trafficking, secretion, and vesicular transport; v) Defense mechanisms; w) Extracellular structures; x) Mobilome: prophages, transposons; y) Nuclear structure; z) Cytoskeleton. Characterization of GO **(B)** and KEGG **(C)** enrichment result for transcriptome assemblies.

In the cluster of orthologous (COG) functions, there are 497 DEGs (Figure 7A). Among them, 72 DEGs were related to secondary metabolite biosynthesis, transportation, decomposition and metabolism. Compared with other functions, the number of differential genes in this group was the largest. The number of differential genes related to carbohydrate transport and metabolism was 67, and the number relating to lipid transport and metabolism was 64 (Figure 7A). Some genes may act in multiple functions. There are three DEGs (0.8%) each involved in four functions; 13 DEGs (2.6%) each involved in three functions; 71 DEGs (14.9%) each involved in two functions and for the remainder (81.7%) each gene had a single function. Among the unigene annotated by GO, 1,289 were significantly differentially expressed, accounting for 5.10%, and the detailed classification is presented in Figure 7B. DEGs were enriched in the biological process module in DNA replication initiation and fatty acid biosynthetic process. In the cellular component module, DEGs were enriched in nucleus, extracellular region, host cell nucleus, among others. In the molecular function module, DEGs were enriched in DNA binding, metal ion binding, heme binding, among others.

After enriching the differentially expressed genes with the KEGG pathway, 551 differential genes were identified related to 119 pathways (Figure 7C). According to the ranking of the DEGs enrichment ratios, among the top 50 pathways 67.3% regulate material metabolism. The plant hormone signal transduction pathway enriched DEGs the most genes, 44 representing 8.0% of the total. The enrichment of DEGs in the phenylpropane biosynthesis pathway were second. The gene c74197. graph_c0 (0.2%) participates in the regulation of ten pathways simultaneously and the log2FC was 1.2, indicating the gene was upregulated. These include carbon metabolism (Ko01200), citrate cycle (TCA cycle) (Ko00020), glycine, serine and threonine metabolism (Ko00260), glycolysis/gluconeogenesis (Ko00010), glyoxylate and dicarboxylate metabolism (Ko00630), lysine degradation (Ko00310), propanoate metabolism (Ko00640), pyruvate metabolism (Ko00620), tryptophan metabolism (Ko00380) and valine, leucine and isoleucine degradation (Ko00280). The gene c77282. graph_c0 (0.2%) is involved in the regulation of seven pathways simultaneously and the log2FC was 8.0, indicating the gene was upregulated. Both of these two DEGs are involved in the regulation of primary metabolism. Other upregulated genes participated in more than one pathway simultaneously, including seven DEGs (1.3%) with each participating in six pathways, 14 DEGs (2.5%) with each participating in 5 pathways; 3.6% of the total DEGs participating in four pathways, 9.1% of the DEGs participating in 3 pathways, 10.2% of the DEGs participating in 2 pathways, and the remainder (72.9%) participating in a single pathway.

### Prediction of Candidate Genes Involved in Material Metabolism

**FIGURE 8:**
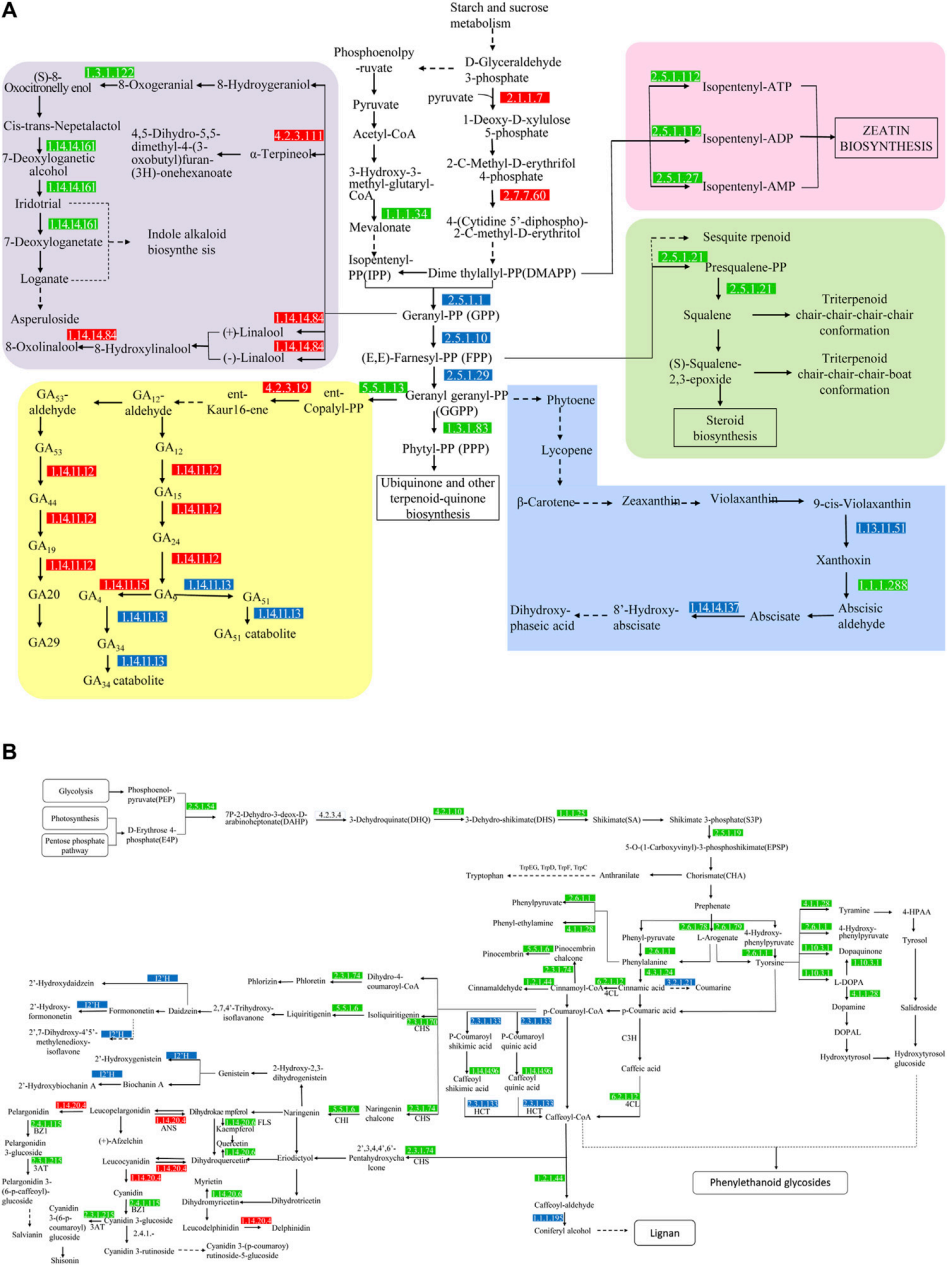
The biosynthetic pathways and genes in the terpenoid **(A)** and flavonoid **(B)** secondary metabolite pathways. The numbers shown in the boxes are the genes/genomes that are enriched in the KEGG pathway that regulate response. The red box indicates that the gene is upregulated, the green box indicates that the gene is downregulated, and the blue box indicates that the genome has up-regulated genes and downregulated genes. The solid line represents a single-step synthesis reaction and the dashed line represents a multi-step synthesis reaction.

**FIGURE 9:**
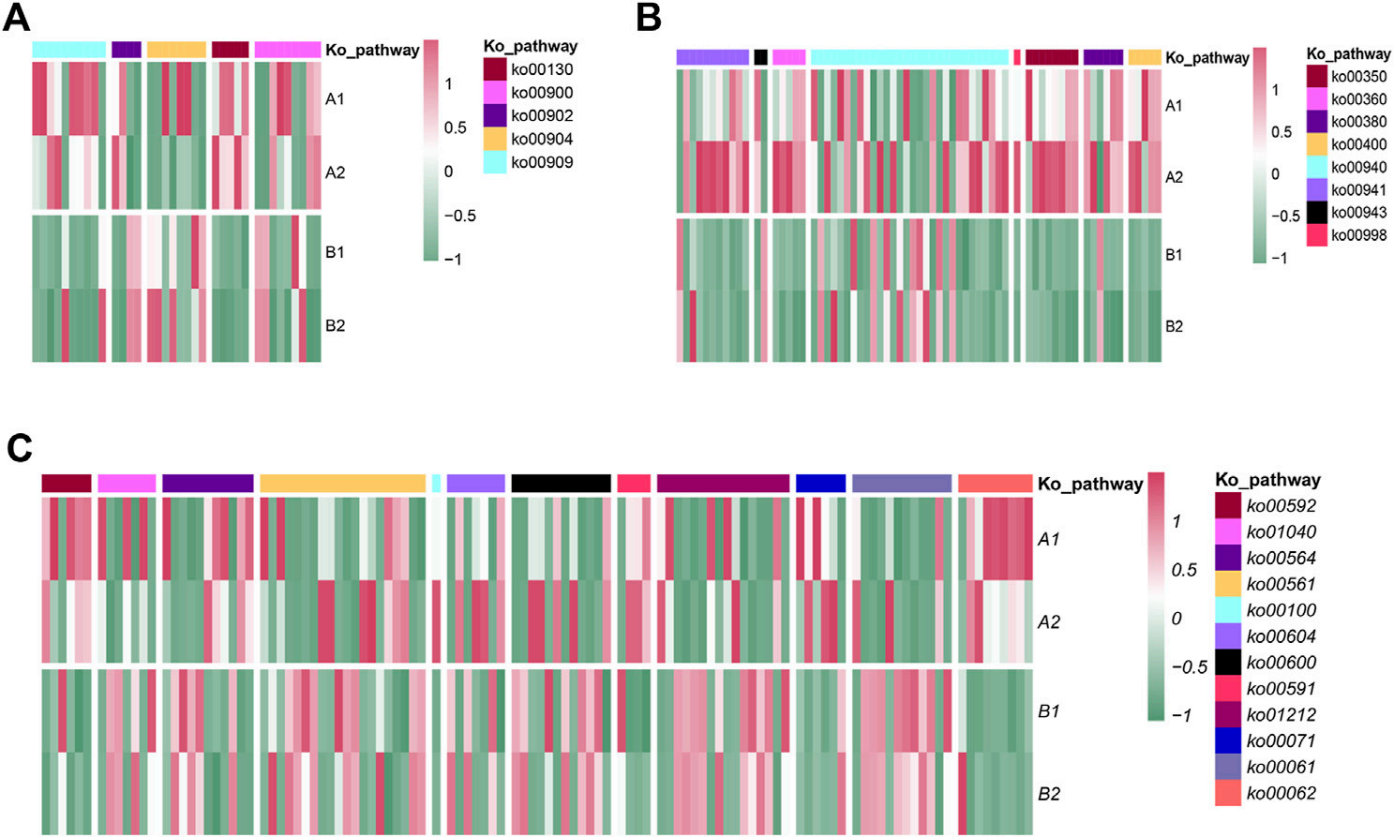
Transcript expression analyses and the biosynthetic pathways of terpenoid metabolites **(A)**, active metabolites **(B)** and lipids **(C)**.

In the KO database annotation, there were 36 differential genes related to terpenoid metabolism (Figures 8A, 9A), including the terpenoid skeleton biosynthesis pathway (Ko00900), monoterpenes (Ko00902), sesquiterpenes and triterpenes (Ko00909), diterpenes (Ko00904), and other terpenes (Ko00130). There were five pathways in total. Isoprene is produced by the mevalonate (MVA) and methyl erythritol phosphate (MEP) pathways in the terpene backbone biosynthesis pathway (Ko00900), and 9 DEGs were involved in regulation of these. The MVA pathway starts from 2 acetyl CoA which is catalyzed by AACT and HMGS to obtain 3-hydroxy-3-methylglutaryl CoA. This substance is catalyzed by 3-hydroxy-3-methylglutaryl-CoA reductase (HMGR, EC: 1.1.1.34) to produce MVA, which undergoes phosphorylation and decarboxylation to produce Isopentenyl-PP (IPP). HMGR is upregulated during plant growth and promotes the formation of terpenoid skeletons through the MVA pathway. The MEP pathway, which starts with a condensation reaction between pyruvate and glyceraldehyde-3-phosphate followed by a condensation reaction catalyzed by 1-deosy-d-xylulose-5-phosphate synthase (EC:2.2.1.7), generates 1-deoxy-d-xylulose-5-phosphate, 2-C-methyl-d-erythritol 4-phosphate in the presence of 2-c-methyl-d-erythritol 4-phospphate cytidylytransferase (EC:2.7.7.60) produces 4-(Cytidine 5′-diphospho)-2-C-methyl-d-erythritol. Both genes were upregulated. Dime thylalllyl-PP (DMAPP) was generated after a four-step transformation. IPP and DMAPP are isomers, which are in equilibrium and are the source of isoprene units. DMAPP and IPP are catalyzed by EC:2.5.1.1 to generate geranyl-PP (GPP). GPP combines with an IPP to generate (E, E)-famesyl-PP (FPP) under the catalysis of EC: 2.5.1.10. FPP will form geranyl geranyl-PP (GGPP) under the catalysis of EC: 2.5.1.29 with another IPP. IPP, DMAPP, FPP, GPP, GGPP, and other substances are the starting materials to produce terpenoids with different structures and are the backbone of terpenoids. GPP is the precursor of monoterpenes and enters the monoterpenoid biosynthetic pathway (Ko00902). FPP is the precursor of sesquiterpenes and triterpenes and enters the sesquiterpene and triterpenoid biosynthesis pathway (Ko00909). GGPP is a diterpene. The precursors of tetraterpene and tetraterpene enter the diterpene biosynthesis pathway (Ko00904) and the carotenoid biosynthesis pathway (Ko00906), respectively. Gibberellins (GAs) are diterpenoids, and there are five genes involved in their synthesis in the Ko00904 pathway. GGPP is regulated by the gene ent-copayl diphosphate synthase (CPS, EC: 5.5.1.13) in the plastid to generate ent-copalyl-PP, and its expression was downregulated and then regulated by the gene ent-kaurene synthase (KS, EC: 4.2.3.19) to generate ent-Kaur-16-ene. KS expression was upregulated. Ent-Kaur-16-ene undergoes a multi-step enzymatic reaction in the endoplasmic reticulum to generate GA12-aldehyde. GA12-aldehyde is regulated by different genes to generate GAs. GA_12_ produces GA_24_ and GA_9_ under the regulation of EC: 1.14.11.12, GA_9_ produces GA_4_ under the regulation of EC: 1.14.11.15, and GA_4_ is regulated by the gene EC: 1.14.11.13 to synthesize GA_34_. Among the GAs on FF, the content of GA_36_ was the highest, and it is metabolized by two pathways, GA_24_ and GA_37_, and can generate GA_4_ under the action of P450 enzymes. GA_8_ and GA_8_-catabolite are metabolites of GA_1_, and EC: 1.14.11.13 are genes that regulate the metabolism of active GAs. In the Ko00906 pathway, GGPP generates carotene, lutein, and other tetraterpenoids through a series of complex regulatory reactions. Carotene and lutein can absorb light energy and participate in photosynthesis and abscisic acid is a signal of seed maturation and stress resistance. Hormones are synthesized via carotenoid metabolism. Differential genes EC:1.14.14.137, EC:1.13.11.51 and EC: 1.1.1.288 are involved in the regulation of abscisic acid metabolism (Figure 8).

Flavonoid synthesis of secondary metabolites (Figure 8B) begins with d-erythrose 4-phosphate (E4P) and phosphoenolpyruvate (PEP). E4P comes from the pentose phosphate pathway and PEP comes from the glycolysis pathway. The two undergo a four-step enzymatic reaction to generate shikimate acid (SA) which is catalyzed by shikimate kinase (AroK/AroL), 5-enolpyruvate shikimate-3-phosphate synthase (AroA), and chorismate synthase (AroC) to generate chorismate (CHA). CHA is a branch point, l-phenylalanine (L-Phe), l-tyrosine (L-Tyr), l-tryptophan (l-tryptophan, L-Trp) of the aromatic amino acids participate in different branch pathways. PheA catalyzes the production of phenylpyruvate, which is catalyzed by amino acid transaminase to obtain L-Phe. TyrA catalyzes the conversion of pre-benzoic acid to 4-hydroxyphenylpyruvate, which is then catalyzed by transaminase to obtain L-Tyr. The synthesis pathway of tryptophan is more complicated, starting with chorismate, and generating L-Trp under the catalysis of TrpEG, TrpD, TrpF, TrpC, TrpA and TrpB. l-phenylalanine is produced by the action of phenylalanine lyase, hydroxylase, and CoA ligase to produce 4-coumayl-CoA, which is produced by chalcone synthase (CHI) and flavanone 3-hydroxylase (F3H) to form dihydroflavonoids. Dihydroflavonols are important precursors of other flavonoids. They are catalyzed by flavonol synthase (FLS), dihydroflavonol 4-reductase (DFR), and anthocyanidin synthase (ANS). The flavonols, anthocyanins, and flavonoids are produced separately. These three substances undergo methylation, glycosylation and acylation modification to form flavonoids with diverse structures. Five DEGs annotated with the phenylalanine acid metabolism pathway (Ko00360) were all down-regulated (Figure 9B). Phenylalanine ammonia-lyase (PAL, EC: 4.3.1.24), functional aspartate aminotransferase and glutamate/aspartate-prephenate aminotransferase (PAT/AAT, EC: 2.6.1.1) and aromatic-l-amino-acid/L-tryptophan decarboxylase (EC: 4.1.1.28) inhibit the conversion and metabolism of phenylalanine, respectively, and inhibit its conversion to cinnamate, phenylpyruvate and phenethylamine. There are eight genes associated with enrichment in the tyrosine metabolism pathway (Ko00350). These genes (EC: 4.1.1.28, EC: 2.6.1.1, EC: 1.10.3.1 and EC: 4.1.1.28) inhibit the conversion of tyrosine to tyramine, hydroxyphenylpropionate, dopamine, and dopaquinone. The flavonoid biosynthesis pathway (Ko00941) is related to 11 genes, except for the upregulation of anthocyanidin synthase (ANS, EC: 1.14.20.4), chalcone synthase (CHS, EC: 2.3.1.74, 2.3.1.170), shikimate O-hydroxycinnamoyl transferase (HCT, EC: 2.3.1.133), chalcone isomerase (EC: 5.5.1.6), and FLS (EC: 1.14.20.6) were all downregulated to varying degrees. In the anthocyanin synthesis pathway (Ko00942), anthocyanidin 3-O-glucosyltransferase (BZ1, EC: 2.4.1.115) and anthocyanidin 3-O-glucoside 6″-O-acyltransferase (3AT, EC: 2.3.1.215) inhibit geranium glycosylation and acylation of vitamins, anthocyanins and delphiniums. In the flavonoid synthesis pathway (Ko00943), CYP81E (EC: I2′H) regulates the hydroxylation of daidzein, formononetin, pseudobaptigenin and genistein. The gene c82894. graph_c0 was upregulated, and the gene c72877. graph_c0 was downregulated.

## Discussion

In the present study, the lipid composition of FF was systematically determined using liquid-phase mass spectrometry. For the first time, it was determined that the FF contains 5,802 lipids and that the content of lipid components with pharmacological activity was relatively low. Comparing the lipid changes of FF during the two growth periods in May and July, the content of 139 lipid types changed significantly. Different lipid secondary metabolites are mainly concentrated in pregnenolones and polyvinyls, among which the content of chalcone, dihydrolone, flavanone, flavonol and mactraxanthin 3-(5Z,8Z,11Z,14Z, 17Z-eicosapentaenoate) 3′-palmitoleate increased, while the content of afzelechin 7-O-β-d-apiofuranoside, 4β-(2,4-dihydroxy-3-methoxyphenyl) fisetinidol, and others decreased. Transcriptome analysis of FF detected 92,294 unigenes and 1,533 DEGs. There were 551 DEGs in the KO annotation, which were enriched in 119 KEGG pathways. Plant signal transduction, interaction with pathogens, and phenylpropane biosynthesis pathways were the most enriched among the DEGs. Based on these results, the present study analyzed the lipid metabolism of FF to explore the regulatory mechanism of terpenoids and flavonoids during the growth process.

FF contains monoterpenoids, sesquiterpenoids, diterpenoids, triterpenoids and tetraterpenoids. Dong et al. (2017) found that the average content of volatile oil in FF was as high as 3.27%, containing α-pinene, β-pinene, limonene, phellandrene, cymene, linalool and terpine-4-ol (Sun et al., 2018; Yang et al., 2014). The results of the present study show that FF contains rosin alcohol, fumagillin, hydroxy camphor, and other substances, as well as plant hormones such as abscisic acid gibberellin. The terpenoid structural unit is transformed from the MVA and MEP pathways to generate IPP and its isomer DMAPP. The present study found that the synthesis of the terpenoid skeleton of FF is dominated by the MEP pathway and the pathway is regulated by genes EC: 2.2.1.7 and EC: 2.7.7.60. Plant hormones are closely related to the growth process of plants. ABA, also known as dormant or abscisin, causes dormancy of buds, shedding of leaves and inhibition of cell growth. Mass spectrometry detected no significant difference in the content of 7′-hydroxyabscisic acid and (+)-8′-hydroxyabscisic acid. Genes EC: 1.1.1.288, EC: 1.14.14.137 and EC: 1.13.11.51 jointly regulate the metabolism of abscisic acid, which may be related to the growth and development stage of FF when harvested. Abscisic acid is balanced under the dynamic regulation of the three genes state. ABA is synthesized by carotene, and the content of carotene decreases significantly, possibly due to the production of abscisic acid. Gibberellin is a diterpenoid compound with diverse structures, some of which are easily soluble in water and only a few substances have intrinsic activity controlling all aspects of plant growth and development (including seed germination, stem elongation, leaf expansion, flowering and seed development) (Hedden, 2020). GA_1_, GA_3_, GA_4_ and GA_7_ have biological activity. The 6 GAs identified were non-biologically active substances. GA_36_ had the most abundant content and is the precursor of active gibberellin GA_4_, which may be transformed into an active state under the control of GA_20_-oxidase (GA20ox, EC: 1.14.11.12) and GA_3_-oxidase (GA3ox, EC: 1.14.11.15). At the same time, GA_8_ and its metabolites, which are the metabolites of GA_1_, may be metabolic genes in (EC: 1.14.11.13), the gene c83901. graph_c3 was upregulated to promote the catabolism of active GAs and c63052. graph_c0 was downregulated to inhibit the decomposition of active GAs. In short, gibberellin has a high content in July in Qingqiao, and its metabolism was active, which fits with it being an important substance for fruit development.

Flavonoids play are important for attracting insect pollinators, resisting ultraviolet rays, and enhancing disease resistance (Onkokesung et al., 2014) in plants (Agati et al., 2012). Flavonoids have various biological activities (Ullah et al., 2020) such as antibacterial, antiviral, antitumor, anti-inflammatory, and hypoglycemic activity (Cazarolli et al., 2006). Studies (Hoshino et al., 1999) have reported that the antibacterial effects of certain flavonoid complexes are related to their oxidative division of DNA or damage to bacterial cell membranes. Wang et al. (2004) found that baicalin inhibited HIV-1 induced syncytium formation and HIV-1 p24 antigen and RT product produced antiviral effects. Pereira et al. (2007) found that naringin can significantly reduce the nitrite content and the amount of inflammatory cells in the macrophage inflammation model (Yang et al., 2015; Nishioka et al., 1998). Flavonoids are based on the C6-C3-C6 structure, and include anthocyanins, flavonoids, flavonols and isoflavones. Pinocembrin, naringenin and eriodictyol are the precursors of other flavonoid derivatives. There are some key enzymes in the *de novo* synthesis of flavonoids. ACCase (acetyl CoA carboxylase) has a wide range of functions, which can guide the flow of carbon from photosynthesis to primary and secondary metabolites. Among these enzymes, MF (multifunctional) guides the biosynthesis of very long-chain fatty acids and flavonoids in the cytoplasm. PAL is the entrance enzyme of phenylpropanoid biosynthesis (Sun et al., 2020). Hu et al. (2011) used a competitive inhibitor 2-aminoindan-2-phosphonic acid (AIP) to specifically inhibit PAL activity in *Cistanche* suspension cells and found that the content of phenylethanol glycosides was significantly reduced. In the phenylpropane pathway, CHS is a key enzyme in the synthesis of flavonoids, leading the central pathway of phenylpropane metabolism to the branch of flavonoid synthesis. The results of the present study showed that when the expression of anthocyanidin synthase (ANS, EC: 1.14.20.4) was upregulated, afzelechin 7-O-β-d-apiofuranoside was significantly reduced. This result may be due to the conversion of afzelechin from leucopelargonidin, and ANS can promote the metabolism of leucopelargonidin into dihydrokaempferol and pelargondin, which indirectly leads to a decrease in the synthesis of afzelechin. Perhaps ANS is an inhibitory enzyme of afzelechin synthesis. In addition, the content of quercetin 7,3′,4′-trimethyl ether 3-rhamnoside (quercetin 7,3′,4′-trimethyl ether 3-rhamnoside) increased significantly, and there were two influencing factors. On the one hand, the upregulation of ANS expression promotes the conversion of leucocyanidin to dihydroquercetin, which is the precursor of quercetin 7,3′,4′-trimethyl ether 3-rhamnoside. On the other hand, the downregulation of flavonol synthase (FLS, EC: 1.14.20.6) (Park et al., 2019) inhibits the oxidation reaction of dihydrokaempferol, dihydroquercetin and dihydromyricetin. FLS is the key enzyme for the conversion of dihydrokaempferol, dihydroquercetin, and dihydromyricetin to pelargonidin, quercetin, and myricetin. The significant reduction of myricetin 7-glucoside again confirms the effect of FLS. Park et al. isolated the OsFLS gene from rice and showed that the OsFLS gene has both flavonol synthase and flavanone 3-hydroxylase activity, which is consistent with the results of the present study.

There are also pharmacologically active substances in FF, such as phenethanol glycosides and lignans, which originate from the shikimic acid pathway and are homologous to flavonoids (Sun et al., 2018). The results showed that the phenylalanine, tryptophan, and tyrosine synthesis pathway (Ko00400), phenylalanine metabolism pathway (Ko00360), the tyrosine synthesis pathway (Ko00350) and the phenylpropane synthesis pathway (Ko00940), and the activity of anthocyanin synthesis pathway (Ko00942), both peaked and declined. The downregulation of these pathways is closely related to the synthesis of active secondary metabolites such as phenethyl alcohol glycosides, lignin, and flavonoids indicating that the accumulation of secondary metabolites reaches the peak during the maturation period of FF in July, when the crop is ready to harvest. The chemical composition and pharmacological effects of Laoqiao and Qingqiao harvested 1 month later differ greatly. Cui et al. (2010) found that the different maturity of FF determines the concentration of chemical substances. Jia et al. (2015) found that forsythin A, forsythin C, cornoside, rutin, forsythin and the gallic acid content is higher than Laoqiao. Bao et al. (2017) found that the anti-tumor activity of Laoqiao on B16-F10 mouse melanoma was significantly weaker than that of Qingqiao. Over time the content of secondary metabolites in FF will gradually decrease and the related pharmacological effects will gradually weaken. During harvesting and processing of FF, a special treatment called “shaqing” is required before Qingqiao is used as a medicine. Shaqing, via steaming, boiling and other processes can destroy the metabolic enzymes (Wu et al., 2019) in FF, so that the active substances are retained. Laoqiao is used as medicine only when dried, which is one of the reasons why Laoqiao and Qingqiao have different active ingredients and effects. The best period for harvesting medicinal plants should be the peak period of synthesis and accumulation of secondary metabolites. Traditional Chinese medicine classics and the Chinese Pharmacopoeia record July to August as the harvesting period of Qingqiao. The transcriptome results of FF in July showed that the DEGs of secondary metabolites biosynthesis, transportation, decomposition and metabolism are the most active at that time. This period is the peak of secondary metabolite accumulation, which proves that the harvesting of Qingqiao from July to August is rational for maximum medicinal benefit.

## Conclusion

In the present study, ultra-performance liquid chromatography-mass spectrometry technology was used to compare the lipid composition and content differences of FF at different growth stages. The correlation between lipid changes in secondary metabolites and differential gene expression was analyzed. Preliminary analysis revealed the biosynthetic mechanism of the main secondary metabolites of FF, indicating that the terpenoid abscisic acid may be in the dynamic regulation of genes EC: 1.1.1.288, EC: 1.14.14.137 and EC: 1.13.11.51. In the synthesis of gibberellin, GA20-oxidase (GA20ox, EC: 1.14.11.12) and GA3-oxidase (GA3ox, EC: 1.14.11.15) catalyze the production of active GAs, and EC: 1.14.11.13 is the metabolic enzyme of active GAs. The synthesis of flavonoids in FF, MF, PAL, CHS, ANS, FLS, and others are all key enzymes. These results provide references for the analysis, development and utilization of the medicinal components of *F. suspense.* In addition, the results of the present study showed that July was the peak period for the accumulation of secondary metabolites in *Forsythia suspensa,* which provids new scientific evidence for the basis of traditional FF harvesting and processing methods.

## Data Availability

The datasets presented in this study can be found in online repositories. The names of the repository/repositories and accession number(s) can be found below: Mass spectrometry data has been uploaded to EBI Metabolights, accession number MTBLS3327. Sequencing data has been uploaded to NCBI SRA BioProject, accession number PRJNA756578.

## Supplementary Material

Supplementary Figure S1 Statistical table of gene expression correlation (square of Pearson’s correlation coefficient) of pairwise samples.

Supplementary Figure S2 Characteristics of differential lipid molecules with OPLA-DA and heatmap.
