# Supplementary figures and images for: Transcriptomic and Lipidomic Analysis of Lipids in Forsythia suspensa
Source: Front Genet. 2021 Oct 26;12:758326. doi: 10.3389/fgene.2021.758326 (PMC8575889; doi:10.3389/fgene.2021.758326)

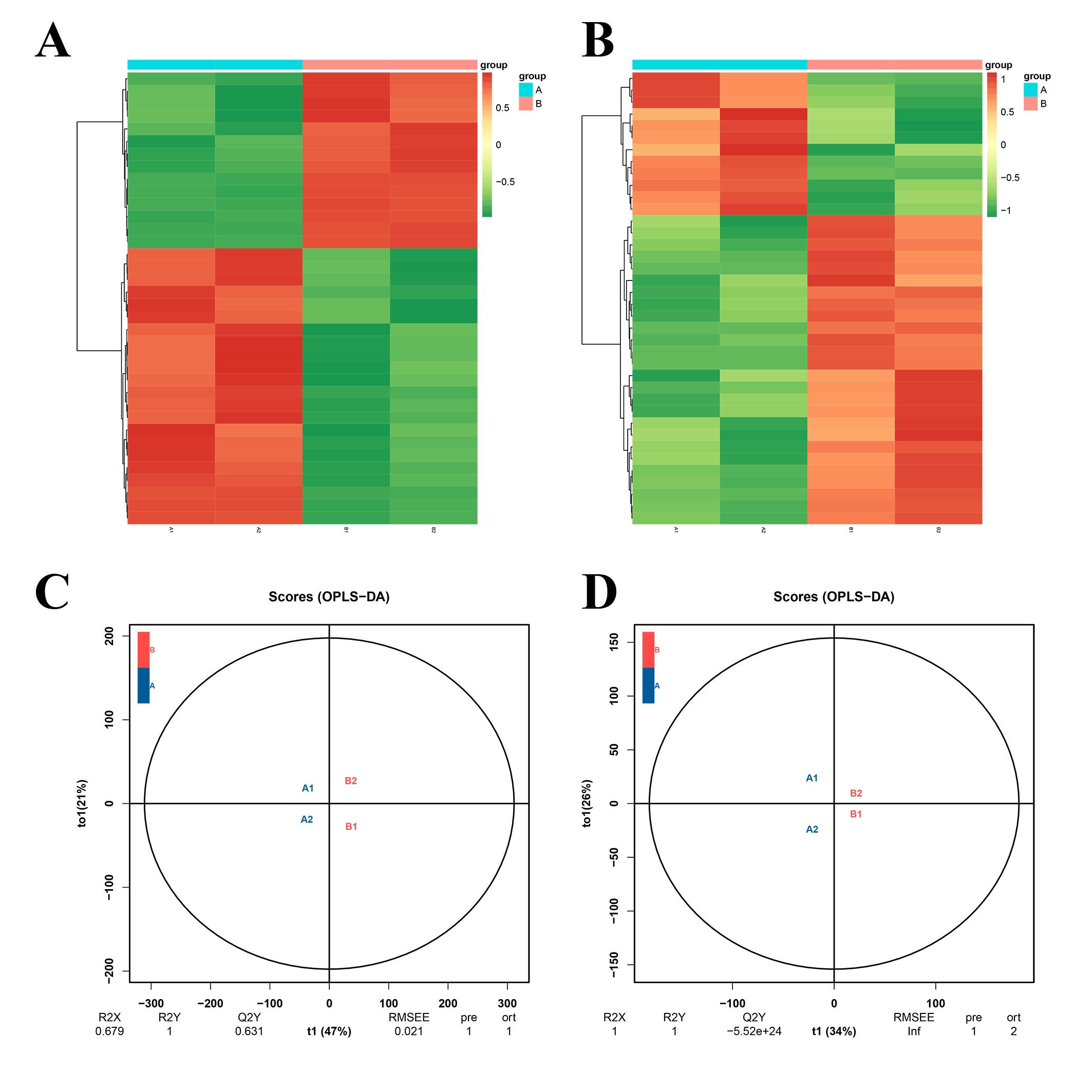

Supplement: Supplementary file 2 [file Image2.TIF]

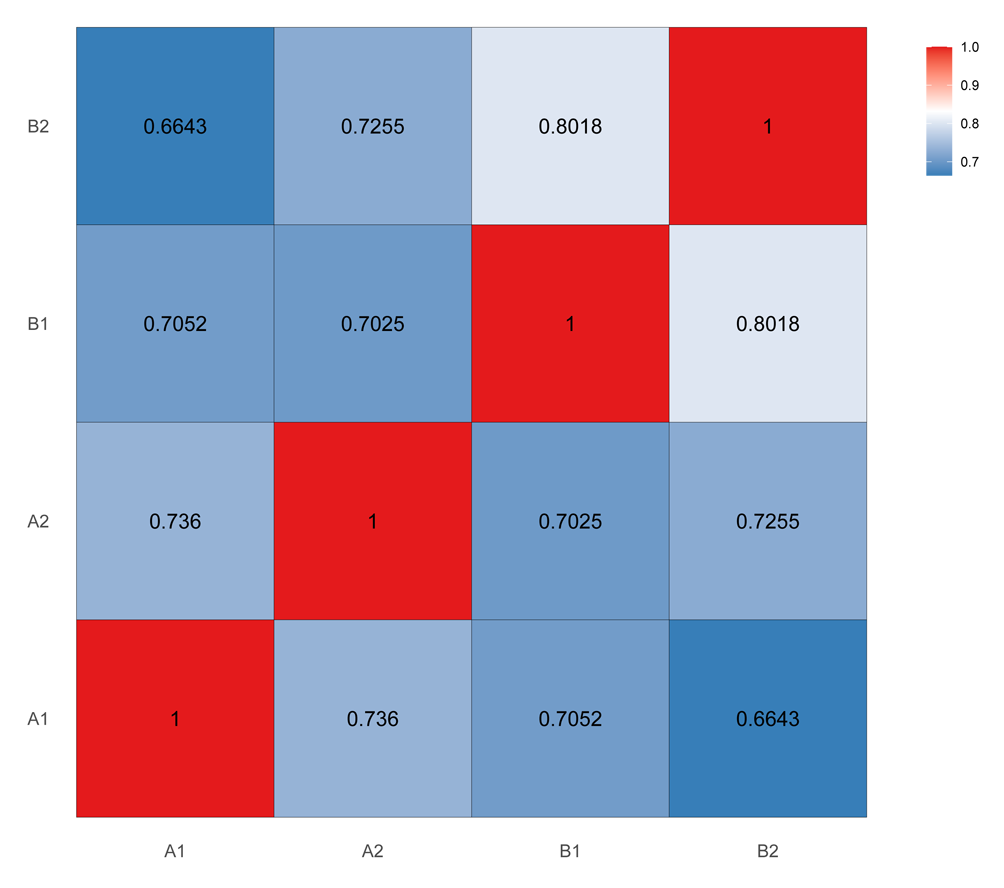

Supplement: Supplementary file 3 [file Image1.TIF]
